# The Ohio State University Dream Team: Innovation for Well-being fellowship and coaching program

**DOI:** 10.1017/cts.2023.558

**Published:** 2023-05-22

**Authors:** Taura L. Barr, Shannon Gillespie, Audra Hanners, Lisa Militello, Christa Newtz, Susan Thrane

**Affiliations:** The Ohio State University College of Nursing, Columbus, OH, USA

**Keywords:** Well-being, innovation, nurse coaching, faculty development, retention

## Abstract

**Background:**

Nearly 70% of faculty experience very high levels of stress. Integrative Nurse Coaching (INC) can help by assisting clients in establishing goals and embarking on new lifestyle behaviors that help to decrease perceived stress, achieve work life integration, and enhance life satisfaction. Our goal was to evaluate a faculty coaching and fellowship program to support faculty well-being while developing innovation competency.

**Methods:**

We employed an INC paradigm to coach five faculty to build confidence and competence in innovation and enhance well-being. We offered monthly group and individual coaching and used a qualitative research thematic analysis to determine themes important for the fellow and group experiences, identify outcomes, and create recommendations for the future.

**Results:**

We identified the following themes as outcomes for our program: (1) enhanced connection, comradery, and support; (2) increased confidence and competence in navigating academia; (3) shift from a fixed mindset to an innovation mindset; and (4) increased ability to identify and manage stress and burnout. Fellows also experienced a shift from focusing on individual needs to addressing the needs of the community at the college.

**Conclusion:**

Nurse coaching is an effective strategy to address faculty stress and burnout. Additional research is needed to evaluate the Innovation for Well-being faculty fellowship program and its impact on the academic community.

## Introduction

The academic environment is demanding. Meeting teaching, research, practice, and service expectations in environments where there are faculty shortages is challenging. Many junior faculty reach a point of frustration and burnout, unless they learn how to effectively manage teaching, research, and practice priorities in a sustainable way. Estimates suggest that more than 1/3 of faculty are burnout or experiencing symptoms of burnout [1]. This contributes to high turnover and poor student experiences, as well as poor faculty health and well-being.

Innovation fills the gap between what is known and what is needed. Innovation produces a product, process, change, or outcome by maximizing existing or adding new value. Innovation can be viewed as both a noun and a verb, and more than anything is a mindset or way of approaching challenges and opportunities with a new way of thinking. When innovation competency is enhanced, well-being increases, through a bidirectional relationship between innovation competency and well-being [2]. Innovation competencies like resiliency, creative thinking, risk taking, visioning the future, courage, and teamwork are known to enhance well-being. Thus, we hypothesized that a coaching program designed to enhance innovation competency would also improve faculty well-being through this bidirectional relationship. We created the Innovation for Well-being faculty fellowship and coaching program and tested its impact on faculty well-being, innovation competency, and efforts to enhance a culture of innovation at our college. Creating communities of innovation through coaching and mentoring is a novel way to address the well-being of faculty in addition to enhancing the research, teaching, service, and practice programs in the academic environment.

Integrative Nurse Coaching (INC) is a subspecialty of nursing that places clients at the center of their lives and views people as integrated whole beings [3]. INCs act as guides to assist clients in establishing goals, embarking on new lifestyle behaviors, and achieving work life integration and satisfaction. The INC coaching process seeks to introduce new narratives into the mind, so new behaviors result. When being coached by an INC, they work specifically to help shift your mindset and equip you with the tools and resources to take ownership of your own health and well-being. A tool INCs use to open people to a wider perspective and their creative potential is awareness. Awareness includes your beliefs, actions, values, habits, and behaviors, and awareness practices are reflective tools that enable the client to take note of who they are and what they want to accomplish.

In 2019, our college conducted a survey to identify the top priorities for faculty and students regarding innovation. Out of 180 responses, the top three resources faculty requested to support their everyday work were: mentoring and coaching (25%); education (25%), and design thinking (20%). More specifically, faculty expressed an interest in being able to better understand how to implement innovation-based practice into their own programs of research, teaching, and practice. Respondents emphasized that they want help and guidance making this information personal and meaningful to them. This is where coaching can be particularly useful.

Thus in 2020 during the pandemic, we created and pilot tested a 1-year Innovation for Well-being fellowship and coaching program to meet the request of our faculty while also addressing innovation competency and faculty well-being. In our program, which we collectively called *the Dream Team*, we employed an INC paradigm to educate and coach five junior faculty to build confidence and competence in innovation to enhance well-being. We also helped each faculty member navigate the faculty role and transition from a self-focused lens to a group focused lens, to equip them with the skills to coach one another and utilize what they learned in the program to enhance their work community during the pandemic. We offered monthly group and individual coaching calls. Group coaching is highly effective in addressing insufficient knowledge, lack of self-confidence, lack of support from family/friends, physical impairments, and creating a sense of social support, where everyone learns from one another [4]. Group is also a great way to create faculty self-care plans, to commit to working in a way that is restorative and in alignment with who you are limiting the effects of moral injury. Group helps participants identify practices that are refreshing enabling participants to choose individualized strategies that allow an internal rhythm and presence within.

In this program, the intent was to create a community of innovators that support one another and learn from one another, while ultimately equipping the participants with the skills and knowledge to coach one another using the Theory of Integrative Nurse Coaching (TINC) method. Thus, the primary objective of the Dream Team was to coach junior faculty to align their work with who they are while enhancing innovation competency and well-being to prevent and manage faculty burnout during the pandemic.

## Methods

The intervention is a 1-year faculty fellowship and coaching program based on the TINC model that includes small group and one-on-one coaching to build innovation competency and enhance well-being.

*Theoretical Framework for the Program:* We used the TINC to guide the implementation of the faculty fellowship program [3]. The TINC is based heavily on healing, the patterns of knowing, and theoretical niches of meaning. Through a self-discovery process, nurse coaches help clients build confidence and competence to transform and seek life and work integration, meaning, and purpose. Healing is an emergent process that brings together one’s individual aspects within a collective community. The nurse coach is a healing presence open to the moment, allowing the individual and group to lead the conversation and new insights to emerge. TINC components include self-development, integral perspectives and change, integrative lifestyle, health and well-being, awareness and choice, and listening with HEART (healing, energy, awareness, resiliency, and transformation). The TINC guided all individual and group conversations.

We based our learning activities and trainings on the Holistic Transcendental Leadership Model for Enhancing Innovation, Creativity, and Well-being in Healthcare [5]. This model of Holistic Transcendental Leadership can be leveraged in the healthcare workplace to enhance innovation and creativity, while placing a novel emphasis on the physical, emotional, and spiritual well-being of the individual, group, and organization. The foundation of Holistic Transcendental Leadership lies in the personal awareness and reflection. From this awareness, leaders are then able to go beyond one’s own self-will and self-interest, to the interest of the team, their community, and the world at large.

In addition, we also used the Innovation Competence Model by Pilay and Morris to design innovation competency conversations [6]. This model includes a core set of 19 innovation competencies. Not all competencies were included in the intervention, just those listed in the intervention (10 of these competencies), with a special emphasis on creativity, courage, and teamwork (connection).

**Figure 1.**
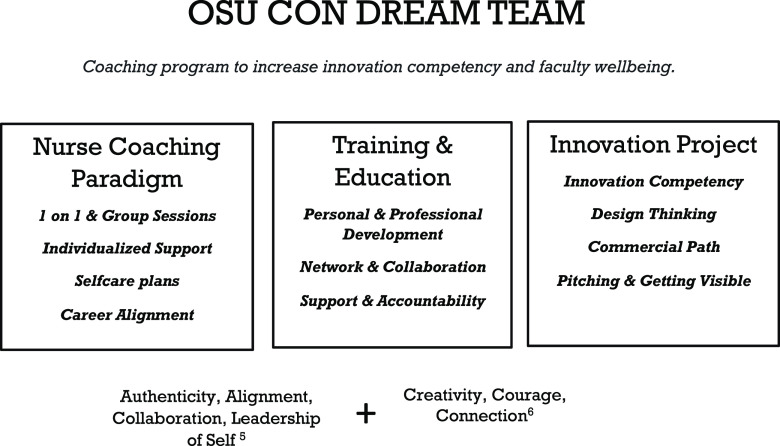
Programmatic details of the innovation fellowship. The Innovation for Well-being fellowship program is built upon the core attributes of Holistic Transcendental Leadership [5] (authenticity, alignment, collaboration, and leadership of self) and innovation competencies [6] (a special emphasis is placed on the core innovation attributes of creativity, courage, and connection). By creating the program from this core set of attributes, we enhance creativity and innovation in the workplace, while also fully supporting the well-being of faculty. Through Hholistic Transcendental Leadership, innovation can be a path to well-being. Through a self-discovery process, we help fellows build confidence and competence in their work to transform and seek life and work integration and bring meaning and purpose to their career. Through these healing relationships and community, creativity, courage, and connection are nurtured, and innovation and healing then emerge from this process that brings together one’s individual aspects within a collective community. To develop these core attributes in our faculty, we built three pillars for the program: (1) nurse coaching to effectively manage faculty goals, mindset and behavior shifts, and facilitate peer learning. (2) Training and education to develop confidence and competence in innovation while also giving faculty the tools to effectively navigate their faculty role within the context of the personal and professional goals. (3) Innovation project to facilitate hands on learning and implementation of the skills and knowledge being learned in the coaching and educational sessions.

*Details of the Intervention:* We employed an INC paradigm based on the concept of Holistic Transcendental Leadership [5] to educate and coach five junior faculty to build confidence and competence in innovation while enhancing well-being. The program is set on three pillars: (1) coaching, (2) education and training, and an (3) innovation project for hands on implementation (Fig. 1).

The Dream Team met monthly as a group to discuss innovation concepts and share strategies for faculty success. Each fellow also met one on one with our primary coach, Dr Barr, to discuss their specific innovation projects and needs surrounding their faculty role. The concepts studied during the year were designed to build and nurture innovation competence and confidence to enhance well-being. Each concept was studied for a month: creativity and curiosity, courage and risk taking, resilience, visioning, identifying opportunities, design thinking, diversity and inclusion, building a team, crucial conversations, self-care and well-being, and most importantly, alignment (Table 1).

**Table 1.** Concepts discussed to enhance innovation and support well-being

Concept	Activities
Alignment	
Creativity and curiosity	Unleashing the inner creator. How to see value in yourself and your ideas. Self-advocacy and assertiveness
Courage and risk taking	Faith in action. Innovation mindset. Strategic risk taking. Pitching your idea
Resilience	Giving light and meaning to challenge, being “antifragile” [9]. Inspired action
Visioning	Creating the future. Brainstorming and mind mapping. Strategic goal setting. Legacy living
Identifying opportunities	Learning to see patterns. Understanding the market. Financing and funding your dream
Design thinking	Fully understanding the problem. Empathy. Designing sustainable solutions. The steps in design
Connection and networking	True collaboration and camaraderie. Leveraging social media. Accountability partners
Diversity and inclusion	Understanding and managing your bias. How to be inclusive. Compassion for others
Building a team	SWOT analysis. Understanding your strengths and weaknesses. What to look for in a partner
Crucial conversations	Asking for what you need. Practice the hard conversations. Non-violent communication. The art of negotiation
Self-care and well-being	Mental health every day. All domains of well-being. Good nutrition. Healthy habits

Educational concepts were identified as foundational to develop Holistic Transcendental Leadership and innovation competency. Each concept discussed every month during the group and individual coaching sessions. Additional activities and opportunities were developed to enhance learning beyond the coaching sessions and fellows decided how deeply they wanted to study each concept based on their personal and professional goals.

### At the Start of the Program, the Fellow

- Identified an innovation project to work on for the year. This was preferably not something new, rather something they wanted to expand upon or take to the next level. The project could be anything, including research, teaching, or practice related, whatever fits the fellows needs.
- Identified a support partner to encourage them on their journey.
- Connected to an accountability partner who was also in the program for support and encouragement.

### During the Program, the Fellow

- Attended 12 monthly online group coaching sessions to offer key leadership skills focused on the basic tenets of Holistic Transcendental Leadership, emotional intelligence, and innovation competency.
- Attended 12 monthly online individual coaching sessions where we discussed the fellows’ chosen innovation project and goals and designed a plan to meet their specific needs. Our goal in these sessions was to pull out the inner innovator and entrepreneur/intrapreneur and equip them with the skills, knowledge, and confidence necessary to infuse a culture of innovation in their education, research, and practice. We also specifically addressed the mental health and well-being needs of the fellow.

*Sample:* This study is considered exempt per IRB regulations; however, all participants were asked to participate and voluntarily agreed. To enhance the fellow experience, encourage robust conversation, and capture preliminary data for analysis, we sought a mix of research, practice, and education faculty. The fellowship is designed for the nurse or clinician scientist, clinician, educator, innovator, creator, and/or intrapreneur/entrepreneur. We recruited faculty through self-identifying an interest in coaching in our 2019 faculty innovation survey. The average size for a group coaching cohort is 5–8 participants; thus, the five faculty who expressed an interest in innovation coaching were contacted by a member of our team and interviewed. All faculty interviewed were invited to participate and all accepted the invitation. No preference or exclusion was granted according to gender or ethnic/racial background.

*Measurement and Analysis:* To examine the subjective experience of fellows in the program, we conducted semi-structured interviews with each fellow between March and June 2021, near the end of their yearly program. Interviews were carried out by one person, and typically lasted 1 hour. Open-ended questions were used, and responses were recorded and transcribed. Transcripts were analyzed using thematic analysis, a qualitative method used for identifying, analyzing, and reporting patterns within data [7]. Through thematic analysis, we determined themes important for the fellow and group experiences, identified outcomes specific to the program, and created recommendations for the future. Fellows also outlined specific barriers, facilitators, and opportunities that can be addressed to enhance the innovation ecosystem in academia.

## Results

Five assistant professors in the College of Nursing participated in this fellowship. Three of these faculty were clinical track faculty, and two were tenure track faculty. Each of them had a different area of expertise, which ranged from pediatrics to gerontology and marginalized populations. Three faculty were in the first 4 years of their faculty role; the other two faculty had been in academia for 5 years or more. One faculty fellow was nearing retirement.

Over the course of 1 year, fellows self-reported higher levels of life satisfaction and well-being. During one-on-one sessions, participants discussed their chosen innovation project and where they needed support. Often, fellows brainstormed areas to “make innovation fit” into tenure and promotion review or challenges with collaboration or engaging their team. During group sessions, everyone helped to brainstorm next steps to advance one another’s idea (e.g., if grant, what is the best mechanism or what needs to be done to get it ready for submission; if manuscript, how to frame it, what journal, if teaching innovation, what are the details). They also generally supported one another and created a sense of community.

The conversations that took up the most time, however, were unrelated to their innovation project. Fellows wanted to share in the experience of being an academic fellow. They wanted to learn how to sustain, maintain, and prioritize their work while supporting their mental health and well-being. Often the participants expressed symptoms of burnout related to their position and career decision. During this time, participants received coaching to understand what may have contributed to their burnout symptoms and how to manage and prevent them now and in the future. During group, participants often shared what they were doing to manage their burnout and peer learning took place during each session. All five fellows shared thoughts on this experience, what worked, what did not work, what they valued the most about the experience, and what they plan to do with their new awareness (Table 2).

**Table 2.** Fellow experiences

**Fellow 1.** “Participation in this program helped me make innovation a priority in my professional life, with the goal of helping patients, families, and communities. The best part of this program was the intentional bringing together of individuals to talk about the importance of and challenges associated with healthcare and educational innovation. A major challenge of such a program is the diversity of innovations, which require working through different processes and program support in varied areas. Time is also often a limiting factor to innovating within the academy, highlighting the importance of building a culture and support system that expects and rewards creativity and perseverance. I most value my college’s transparent commitment to innovation, which is made clear by developing and supporting these types of programs. I will now navigate my entire research career in consideration of design thinking, with the hope of better serving patients, providers, and the entire healthcare sector.” A major challenge of such a program is the diversity of innovations, which require working through different processes and program support in varied areas. Time is also often a limiting factor to innovating within the academy, highlighting the importance of building a culture and support system that expects and rewards creativity and perseverance. I most value my college’s transparent commitment to innovation, which is made clear by developing and supporting these types of programs. I will now navigate my entire research career in consideration of design thinking, with the hope of better serving patients, providers, and the entire healthcare sector.”
**Fellow 2.** “I was honored to participate in this program and thankful to have a safe space to share ideas and discuss challenges. Having a group to help identify, troubleshoot, and problem solve issues that arise not only with the innovation/solution itself, but also overcoming barriers to innovating in an academic setting was incredibly helpful. Although the time commitment was minimal and flexible, time is a very precious commodity for faculty. As such, the time needs to be spent towards outcome metrics valued for faculty (e.g., manuscripts, grants). Validating and sharing ideas with like-minded individuals in the space of innovation and entrepreneurship (I&E). While diverse opinions are often welcome in academia, there can exist an underlying battle between traditional research/scholarship (i.e., NIH/NSF funding) compared to partnering with industry, hackathons, and commercialization efforts. Continue to foster my growth & understanding of I&E through fellowships and collaborations. Recognize that there is a strong presence of innovators within the academic setting that are doing amazing things to trailblaze a path between scholarship and clinical/public to have broad impact.” Having a group to help identify, troubleshoot, and problem solve issues that arise not only with the innovation/solution itself, but also overcoming barriers to innovating in an academic setting was incredibly helpful. Although the time commitment was minimal and flexible, time is a very precious commodity for faculty. As such, the time needs to be spent towards outcome metrics valued for faculty (e.g., manuscripts, grants). Validating and sharing ideas with like-minded individuals in the space of innovation and entrepreneurship (I&E). While diverse opinions are often welcome in academia, there can exist an underlying battle between traditional research/scholarship (i.e., NIH/NSF funding) compared to partnering with industry, hackathons, and commercialization efforts. Continue to foster my growth & understanding of I&E through fellowships and collaborations. Recognize that there is a strong presence of innovators within the academic setting that are doing amazing things to trailblaze a path between scholarship and clinical/public to have broad impact.”
**Fellow 3.** “I’m grateful to have been a part of this innovation workgroup. When I think about what I gained most from this innovation space, I put emphasis on creativity and comradery. For me, innovation is fueled by having a dedicated space and time to be creative. This workgroup provided a space where creativity was welcomed, celebrated, inspired, and supported. Our group members spanned several faculty delineations, levels of teaching/practice and various specialty interest areas. The comradery felt, however, was as cohesive as any group I have been a part of. I attribute this to the seemingly high emotional intelligence levels of the group members. Each person brought their own creative flare to the group, and I loved learning about the projects everyone was working on. The creative resiliency of fellow workgroup members was inspiring and motivated me to keep being persistent with my own projects. The lively discussions and consequent support provided a sense of community during an otherwise isolated pandemic environment that I know will continue beyond this fellowship.”
**Fellow 4.** “The Dream Team enabled me to genuinely connect in a unique safe space. I was able to explore creative ideas unrestrained. I appreciated how much diversity was embraced and celebrated. In this space we were all sitting at the table as equal partners and supporters of our unique niches in nursing not as competitors or seniority ranking etc. It was amazing how the common thread of innovation elevated us above potential discrimination to a place of unity and shared vision of creating a healthier world in our own unique ways. Having group meetings to bounce ideas of each other or learn from each other’s mistakes and success. Sharing resources, contacts, and tips/strategies. In ability to have protected time, reimbursed time or have time invested in innovation recognized towards promotion. Safe space to dream big and connect with others who did the same. It was energizing to help support dreams of others while working on my own. Fully pursue alignment with innovative ideas that I was able to generate and start refining because of this program by bringing these skills learned into my current job role.”
**Fellow 5.** “I’m near retirement, but still have a passion for making a difference. I felt incredibly stifled in my current role and couldnʼt see the next steps to enjoy the remainder of my career. This program changed all of that. I now see what is important to me and understand how to make that happen in my career.”

Each fellow was asked to share their overall experience, what worked well, what could be improved upon, what they valued most, and how this fellowship enhanced their career.

Group Outcomes of the Program: We identified the following outcomes based on group experiences and thematic analysis: enhanced connection, comradery, and an overwhelming feeling of finally being supported. In addition to these themes, fellows also agreed that the fellowship:

- Enhanced their confidence and competence in navigating their faculty role, especially expectations for academic promotion and tenure.
- Gave them a clear understanding of innovation and how to implement innovation in their practice, research, and teaching. Specifically, each fellow was able to identify specific examples of how to include innovation in their daily work and how to present their innovation projects for promotion and tenure review.
- Helped them to manage and prevent symptoms of burnout through legacy living: aligning daily work with long-term goals.

An unexpected outcome that was discussed by the group and noticed particularly by the coach who facilitated the fellowship was that over the course of the year, the fellows found themselves creating potential solutions to enhance innovation and well-being, instead of focusing on all the barriers they were experiencing. They also experienced a transformational shift, from focusing solely on individual needs to addressing the needs of the collective community of the Dream Team and at the college. This was exciting to watch unfold and was incredibly empowering for the individual participants. Instead of blaming or complaining, they began to explore the structures that hindered innovation. They identified ways to improve the collective experience of faculty. Most importantly, they began to coach one another. They reminded each other to stay true to themselves, to prioritize their schedules, and find ways to support their health and well-being, while being innovative and creative in their faculty roles.

*Barriers to Innovation Identified by Junior Faculty:* Conversations about the barriers to innovation often took place in the group session. Solutions were also identified and are presented below.

1. Time and energy: Conversations about time and energy took up a lot of group time. Fellows believe there are too many things vying for their attention in academia and feel as if they are doing the job of three people. Do more with less, was the motto. However, this is an individual perceived barrier and an organizational scheduling conflict. Through effective boundary setting, assertiveness, and prioritization, this perceived barrier can be addressed from both an organization and an individual standpoint. Energy management should be addressed at the program chair and leadership level to set realistic expectations for faculty.

2. Decision fatigue: Fellows often said, “I feel like I’m reinventing the wheel or canʼt find the recourses I need to do my job well.” Participants felt it was hard to “keep up” with what was going on at the college because there was so much going on. And thus, they always felt it was just easier to put their innovations on hold. They often had to do it themselves as opposed to partnering with someone who may have the resources or materials they needed. They found there to be little consistency in where information could be found (documents are shared by email, SharePoint, shared drives, box, canvas, website, WordPress sites, etc.). This can be addressed through enhanced organizational and leadership communication strategies and consistent updates.

3. Staff support unevenly dispersed: Faculty have noticed that staff are either not assigned to junior faculty or if they are, they are often redirected to other more senior faculty who “need them” more than the junior faculty. However, junior faculty are often managing students and other staff and would benefit greatly from having support too. When staff members are split in terms of percent effort between teams, it is extremely challenging to create a supportive environment with buy in if others are asking more of that staff member than they can do with their time. Because junior faculty have limited staff support, they tend to get overwhelmed with tasks and put them off or do not do them at all. However, this may be an individual perceived barrier. More effort is needed to understand how staff are distributed across faculty and whether there are opportunities for junior faculty to have enhanced support for scheduling and administrative tasks.

4. Limited partnerships and collaboration: Faculty felt as if they were all doing things differently even when shared approaches made sense and could be developed and scaled easily. For example, the academic culture is based more on individual success, over team success, which leads to unnecessary competition that compromises community success (e.g., patient recruitment, teaching resources, etc.). Many faculty work in isolation. Innovation is a team sport, thus for innovation to flourish this culture needs addressed, particularly through changes made to promotion and tenure guidelines.

5. Promotion and tenure are individually based: Faculty believe they are not incentivized (and sometimes actively discouraged) from helping each other due to the ways in which they are evaluated (e.g., papers and grants as first or corresponding author is critical for tenure track). When faculty help other faculty on grants and papers, it jeopardizes their individual evaluation criteria. This creates misalignment in faculty, especially since they want to be part of a team and helping others but feel like they are actively encouraged to keep their focus on themselves. Promotion and tenure guidelines should reflect the nature of teams and allow for innovation to flourish.

Facilitators of Innovation: It was often harder to identify facilitators to innovation, but as a group we encouraged one another to look for what is working and build upon this.

1. Structure of Innovation: Faculty felt supported and encouraged by the fellowship. They also were happy just knowing the Center for Healthcare Innovation and Leadership and the innovation studio were there for them to help answer questions, support their individual programs, and assist them in implementing their innovation projects. Formal structures create a framework for innovation success and a place to go for mentoring and support.

2. Opportunities for Reflection: Faculty stated over and over how beneficial it was to “pause” and reflect to determine where they were going. Opportunities like the monthly group coaching calls, the yearly faculty retreat our college hosts, and visioning exercises often employed during our faculty meetings were viewed very favorably and often were a source of significant inspiration for faculty. They wish they had more of these events just to brainstorm and dream to allow their creativity to flourish.

3. Protected time to plan and work on creative and innovative projects. Faculty expressed how grateful they were for this fellowship to have protected time for innovation projects, just like they have protected time for their research. Innovation and research go hand in hand; thus, opportunities to support faculty along the commercialization pathway are an untapped resource for faculty success.

4. Creating a culture of innovation by seeing failure as an opportunity to improve, not to be disciplined. The faculty whose mentors did not point out where they were falling behind in promotion and tenure felt more supported to create and innovate. Failure must be seen as a mechanism for success and encouragement goes a long way.

5. Opportunities for junior faculty to use their creativity for the college, not just their own programs. Faculty are creative by nature and have a lot to offer the college. Creating opportunities for faculty to be creative as a group can enhance culture and strengthen community.

### Potential Solutions to Barriers Identified and Recommendations for the Future of Innovation in Academia

We recently identified best practices for innovation to flourish in the academic environment [8]. Providing ongoing mentoring, support, and coaching is a key foundational approach to launch and sustain innovation initiatives. In our prior studies, we have found that very few schools provide ongoing support and coaching for faculty to fully integrate innovation into their research, practice, or educational initiatives; thus, we see this fellowship as an opportunity to enhance the impact of innovation initiatives in academia. To this end, we have identified five specific opportunities for academic units who are looking to begin a coaching or mentoring program for faculty.

1. Invest in the well-being of junior faculty through coaching to work through perceived and real-time and energy barriers, align who they are with what they do, and create confidence and competence in innovation for tenure and promotion review. Coaching is an effective intervention to address imposter syndrome, juggling challenging work and life schedules, and even helping faculty create realistic and manageable goals. Most faculty are not sure how to position their work to get “credit” for innovation activities, so helping faculty with self-advocacy and self-promotion is important. We have also found faculty to benefit from learning how to be more assertive, setting and keeping healthy boundaries, and addressing perfectionism and self-sabotaging behaviors. Coaching can address all of these in a comfortable safe environment.

2. Address decision fatigue by organizing resources. Faculty suggested that a partnership with marketing and IT and use of a full-time dedicated staff working toward the organization of resources and decisions to use them would be incredibly helpful as they navigate their faculty role. A major investment in the organization of electronic resources and an easy means to track who downloads and uses these resources is highly encouraged – this must be in one place and strategically organized with a process to vet the foundational information (which must be up to date) and the templates that are included (specific content areas with only the top resources provided). As an example, there are different types of advisor meetings each semester – It would be great to see a landing page for advisors of each type of student with an up to date one page go to document of what to expect typically and how to proceed with commonly encountered issues (templates are incredibly helpful and help to save everyone time). Simplicity is key and faculty should not be reinventing the wheel.

3. Reconsider how staff and human resources are used and assigned. This includes staff resources and having those staff with very specific and clear areas of expertise and assignments appointed to specific individuals in terms of who they work for and the expectations for how work is requested and allocated. Appropriate allocation of resources is a necessity.

4. Create team-based shared deliverables (with “shared deliverables” being the critical piece here): Consider the concept of academic team lets. Create a tool to help faculty form those groups in which you measure and match working style, dreams, deliverables, and complimentary but not overlapping skillsets. Being part of a community is important.

5. Review promotion and tenure guidelines to ensure innovation is evaluated appropriately and faculty are supported to engage in innovative activities. This may require faculty awareness and review of the current guidelines to identify where improvements can be made. What is not evaluated simply does not get done.

### Limitations

There were several limitations to this study. First, all the faculty who participated in this fellowship had an interest in both innovation and coaching, and thus self-selected for this fellowship. Thus, all fellows were engaged and excited about this fellowship, which helped to create a great group dynamic. This experience may be different in faculty who are forced to participate in such a fellowship. However, given, what we know about the diffusion of innovation, this is not recommended. This fellowship is focused on innovators and early adopters. Based on the diffusion of innovation theory, these groups of individuals participate in new programs first and then become very influential in helping to spread new ideas and programs to other groups. An additional limitation to this study is that all the faculty who participated were from the same college who very highly supports both innovation and well-being. It is not known, whether the success of the program would be different if faculty were selected from disciplines or departments that create inordinately adverse or non-supportive environments. Lastly, it is possible that the outcomes experienced with this fellowship were influenced by varying personal factors of the fellow. Each of the fellows experienced challenges in their personal and professional lives during the course of this fellowship, but we did not take these into consideration during analysis given the nature of this pilot study. Future studies are needed to address these limitations.

## Conclusions and Next Steps

In this pilot study, we have found nurse coaching to be an effective strategy to address faculty well-being while enhancing innovation competency. Additional research is needed to evaluate the long-term effectiveness of the Innovation for Well-being faculty fellowship program and its impact on the academic community. There are many things that could impact fellow well-being; thus, in the future, we will attempt to capture the fellow’s perception of their life circumstances and opportunities during the fellow interview and via surveys to determine how much the fellowship impacts their overall well-being outside of their life circumstances or environment. Since this is a pilot study, we will also determine the most appropriate confounding variables to study and control in the future. Given the success of the Dream Team, we are expanding our fellowship program to include faculty outside of the college of nursing and beyond the Ohio State University nationally. We aspire to create an international program following the launch of our national cohort.
